# Awareness of Stroke Risk Factors and Warning Signs Among Diabetic Patients in the Aseer Region, Saudi Arabia: A Cross-Sectional Study

**DOI:** 10.7759/cureus.42562

**Published:** 2023-07-27

**Authors:** Khalid M Alkhalifah, Aljohrah M Al Hunaif, Banan S Alghamdi, Reema S Alqhatani, Dalia M Almanea, Alhanouf A Alshahrani, Ghaida M Alhaneef, Zainah Al-Qahtani

**Affiliations:** 1 Unaizah College of Medicine and Medical Sciences, Qassim University, Ar Rass, SAU; 2 College of Medicine, King Khalid University, Abha, SAU; 3 College of Medicine, Albaha University, Albaha, SAU

**Keywords:** diabetes, warning signs, risk factors, complication, ischemic and hemorrhagic stroke, stroke

## Abstract

Objective

This study aimed to determine the awareness of stroke risk factors and warning signs among diabetic patients in the Aseer Region, Saudi Arabia.

Methods

We adopted a cross-sectional study involving 314 participants in the Aseer Region, Saudi Arabia between February 27, 2023, and April 20, 2023. The target population was diabetic patients whose database was obtained from various health institutions. The questionnaire was then distributed to the respondents after obtaining informed consent. The data were analyzed using SPSS Statistics version 27.0.1.0 (IBM Corp., Armonk, NY, USA) to obtain important insights.

Results

The majority of the respondents (69.11%) demonstrated a good level of knowledge, while 30.89% had poor knowledge. A good proportion of the respondents (68.47%) knew about stroke, while 73.89% knew it primarily affects the brain. Most respondents claimed that elderly people were at higher risk of attack (52.55%) and that the younger population was also prone to stroke (64.97%). The respondents identified hypertension (74.52%), smoking (72.61%), diabetes (71.66%), and high blood cholesterol (68.47%) as the main risk factors for stroke. The participants also demonstrated a very good understanding of the warning signs, with difficulty speaking (80.57%) and the weakness or inability to move one-half of the body (85.35%) being the most common signs.

Conclusion

The findings in this study indicated a good level of understanding and awareness of stroke, its risk factors, and warning signs (69.11%). The older respondents and people with a higher level of education demonstrated more knowledge. The two variables, age and education, had a statistically significant relationship with the knowledge of stroke. The common risk factors associated with stroke were identified as hypertension, smoking, diabetes, and high blood cholesterol. The respondents demonstrated good knowledge of the warning signs, with the vast majority asserting that difficulty in speaking, decreased sensation, and weakness or inability to move one-half of the body are common warning signs.

## Introduction

Stroke is the third-most common cause of disability and the second-most common cause of death worldwide [1]. The latest data show that every year, approximately 15 million people have a stroke, which causes more than five million deaths [2]. Major factors related to lifestyle and environment changed and raised the risk and incidence of stroke in Saudi Arabia, a nation that has undergone tremendous development during the previous two decades [3]. In the Kingdom of Saudi Arabia, the prevalence of stroke is 43.8 per 100,000 people, with poor public understanding and awareness serving as contributory causes [4]. Furthermore, 14,000 deaths in Saudi Arabia from all causes were attributed to stroke in 2012 [2]. According to a number of studies, smoking, ischemic heart disease, hypertension, diabetes mellitus, hyperlipidemia, and obesity are the main risk factors for stroke [3]. It is generally established that leading a sedentary lifestyle and spending a lot of time watching TV are risk factors for stroke [5]. If lifestyle and other changeable risk factors were targeted by the community or on an individual basis, it is thought that stroke is a disease that can be prevented [6].

In a recent study, the general Saudi population's knowledge of stroke risk factors and warning signs was evaluated, and it was discovered that 63.8% of participants had low levels of knowledge [7]. More than 50% of the patients in a different study of stroke patients at King Abdulaziz Medical City in Riyadh, Saudi Arabia, were not aware that they were experiencing a stroke. Due to a failure to recognize symptoms and indicators, the majority of them sought medical attention too late [8]. Regarding awareness of stroke among diabetic patients in Saudi Arabia, there was only one study in the Al-Hasa region that showed 56.9% of diabetic patients had poor awareness levels [9].

Consequently, due to a lack of information regarding awareness of stroke among diabetic patients in Saudi Arabia, this study aimed to assess patients' knowledge of stroke symptoms, risk factors, and preventative health practices in the Aseer region. As a result, these patients will have a decrease in their risk of stroke. The study's findings will aid the regional health authorities in developing efficient educational initiatives to raise these patients' levels of awareness. This may eventually lessen the challenges and expenses associated with stroke.

## Materials and methods

Study design

The study design was an observational cross-sectional design.

Study participants

The total recruited number was 909 participants, of whom 314 were diabetic and considered for analysis.

Inclusion and exclusion criteria 

In this study, we included diabetic patients residing in the Aseer Region and adults (18 years old and older) who consented to be part of the study. And excluded non-diabetic minors (less than 18 years old) who refused to participate.

Study sample and setting

Between February 27, 2023, and April 20, 2023, this study was carried out in the Aseer region of Saudi Arabia. A self-administrated questionnaire was used in this cross-sectional, observational study. The study was reviewed and approved by the Research Ethics Committee (REC) of King Khalid University. In order to evaluate the awareness of stroke risk factors, warning signs, and preventive behavior among diabetic patients, a total of 384 participants were needed.

Sample size

The estimated sample size was determined using the formula: ss = (Z2pq)/c^2^, where ss = sample size, Z = 1.96, p = 0.5, q = (1−p) = 0.5, and c = sampling error of 5%. In total, 314 respondents participated. Those who met the inclusion criteria were included, and those who did not meet them were excluded. An online questionnaire was distributed through social media (Twitter, WhatsApp, and Telegram). The agreement to fill out the questionnaire was considered consent to participate in the study.

Data collection

The data were collected by a group of trained data collectors who distributed the questionnaire through social media (Twitter, WhatsApp, and Telegram). The self-administrated questionnaire was divided into three sections. The first section collected demographic data such as the participant’s age, gender, education level, and economic status. The second section is for medical and family history. The third section was for awareness regarding stroke, risk factors, and consequences. After permission, the questionnaire was fully taken from the study published by Elshebiny et al [9]. A knowledge item score of 60% and above was deemed good knowledge, while below 60% was poor knowledge. Above 80%, it was considered very good knowledge.

Statistical analysis

Simple descriptive statistics of the participants' sociodemographic characteristics in the form of frequencies and percentages were calculated and tabulated. For quantitative variables, means and standard deviations (SDs) were reported as measures of central tendency and dispersion, respectively. Fisher's exact tests were applied and interpreted to compare qualitative variables, including the participant’s knowledge of stroke risk factors, warning signs, and preventive behavior. Significance was established at a p-value of 0.05 or less (unless otherwise specified) with a 95% confidence interval. All statistical calculations were performed using SPSS Statistics version 27.0.1.0 (IBM Corp., Armonk, NY, USA).

## Results

A total of 314 respondents participated in the study (Table 1). The sample was composed of 114 males (36.31%) and 200 females (63.69%). Most of the respondents were aged between 18 and 24 years (42.68%), while the least represented group was that aged between 25 and 35 years (10.19%). When the question on marital status was posed, it was determined that most of the respondents were married (53.18%), while the rest were either single, divorced, or widowed (46.82%). It was evident that most of the respondents had attained college-level education (56.69%), while the next common group was those that had attained only secondary education (24.84%). Regarding the area of residence, it was found that most of the respondents resided in Abha (46.18%), others in Khamis Mushait (39.49%), and the rest resided in other areas. Finally, when the income level was assessed, it was determined that the majority of respondents earned less than 3000 SAR per month. The social demographic variables are illustrated in Table 1.

**Table 1:** The social demographic variables of respondents.

Variables	Category	Count	Percentages
Sex	Male	114	36.31%
	Female	200	63.69%
Age	18-24 Years	134	42.68%
	25-34 Years	32	10.19%
	35-49 Years	66	21.02%
	50-65 Years	82	26.11%
Marital status	Single/widowed/divorced	147	46.82%
	Married	167	53.18%
Educational level	Primary	30	9.55%
	Middle	28	8.92%
	Secondary	78	24.84%
	College	178	56.69%
Area of residence	Abha	145	46.18%
	Khamis Mushait	124	39.49%
	Other	45	14.33%
Occupation	Medical field	19	6.05%
	Non-medical field	96	30.57%
	Unemployed	199	63.38%
Income	Less than 3000	149	47.45%
	3000 to 5000	33	10.51%
	5001 to 10000	47	14.97%
	More than 10000	85	27.07%

It was determined that 21.02% of total respondents had hypertension, 16.88% had high cholesterol, and 4.78% had cardiovascular disease (CVD). In terms of smoking behavior, 50 respondents (15.92%) had been smoking regularly in the last year, while the rest (84.08%) did not. Exactly 27.71% of the respondents had a family history of stroke or brain hemorrhage, while only 8.28% had a stroke. In terms of frequency of attack, the majority of those who had a stroke experienced it once (53.84%), as shown in Table 2.

**Table 2:** Individual and family medical history of respondents.

Item	Variable	Count	Percentage
Has your doctor told you that you have any of the following health problems:	Cardiovascular disease	15	4.78%
	High blood cholesterol	53	16.88%
	Hypertension	66	21.02%
	Nothing	180	57.32%
Do you smoke regularly for more than a year?	No	264	84.08%
	Yes	50	15.92%
Has anyone in your family had a stroke (ischemic or bleeding in the brain):	I don't know	50	15.92%
	No	177	56.37%
	Yes	87	27.71%
Have you ever had a stroke (ischemic or brain hemorrhage):	I don't know	21	6.69%
	No	267	85.03%
	Yes	26	8.28%
If yes how many times have you had stroke (n = 26)	Once	14	53.84%
	Twice	8	30.77%
	Three times	3	11.54%
	More than three times	1	3.85%

The results indicate that a total of 125 respondents (68.47%) were aware of stroke. A high proportion of them (73.89%) asserted that strokes affect the brain (73.89%). When the question of sex as a risk factor was asked, most of the respondents (35.67%) did not know whether sex is a predictor of stroke or not. Most of the respondents (52.55%) believed that the elderly or people above 50 years were more susceptible to stroke. Most of the respondents also asserted that young people were at risk too (64.97%). Table 3 presents the general awareness regarding stroke.

**Table 3:** General awareness items about stroke.

Item	Variable	Count	Percentage
Do you know the term stroke?	No	99	31.53%
	Yes	215	68.47%
Stroke is a disorder that primarily affects:	Blood sugar	8	2.55%
	I don't know	52	16.56%
	The brain	232	73.89%
	The heart	22	7.01%
Do you think its risk rate:	Equal	64	20.38%
	Higher in females	45	14.33%
	Higher in males	93	29.62%
	I don't know	112	35.67%
Which of the following age groups is more likely to have a stroke?	30-50 Years old	72	22.93%
	I don't know	66	21.02%
	Less than 30 years old	11	3.50%
	More than 50 years	165	52.55%
Can younger people have a stroke?	I don't know	92	29.30%
	No	18	5.73%
	Yes	204	64.97%

Most respondents showed good knowledge of the common risk factors associated with stroke (Table 4). More than 70% of the respondents were aware that hypertension, smoking, diabetes, and high blood pressure were risk factors.

**Table 4:** Awareness of common risk factors associated with stroke.

Risk factor	Yes	%	No	%	I don’t know	%
Hypertension	234	74.52%	31	9.87%	49	15.61%
Smoking	228	72.61%	40	12.74%	46	14.65%
Diabetes	225	71.66%	24	7.64%	65	20.70%
High blood cholesterol	228	68.47%	28	8.92%	58	22.61%
Vascular rapture	156	49.68%	60	19.11%	98	31.21%
Vascular blockage	236	75.16%	20	6.37%	58	18.47%
Stress	174	55.41%	60	19.11%	79	25.16%
Anxiety	182	57.96%	49	15.61%	83	26.43%

Table 5 below indicates that there was a very good knowledge of the clinical features of the stroke. More than three-quarters of the respondents (75%) had good knowledge of the warning signs associated with stroke.

**Table 5:** Awareness of warning signing associated with stroke.

Clinical features	Yes	%	No	%	I don’t know	%
Difficulty speaking	253	80.57%	19	6.05%	42	13.38%
Weakness or inability to move one-half of the body	261	83.12%	15	4.78%	38	12.10%
Decreased sensation or inability to feel things	239	76.11%	22	7.01%	53	16.88%
Low vision	243	77.39%	20	6.37%	51	16.24%
Do you think that people can reduce the risk of stroke	268	85.35%	7	2.23%	37	11.78%

**Figure 1:**
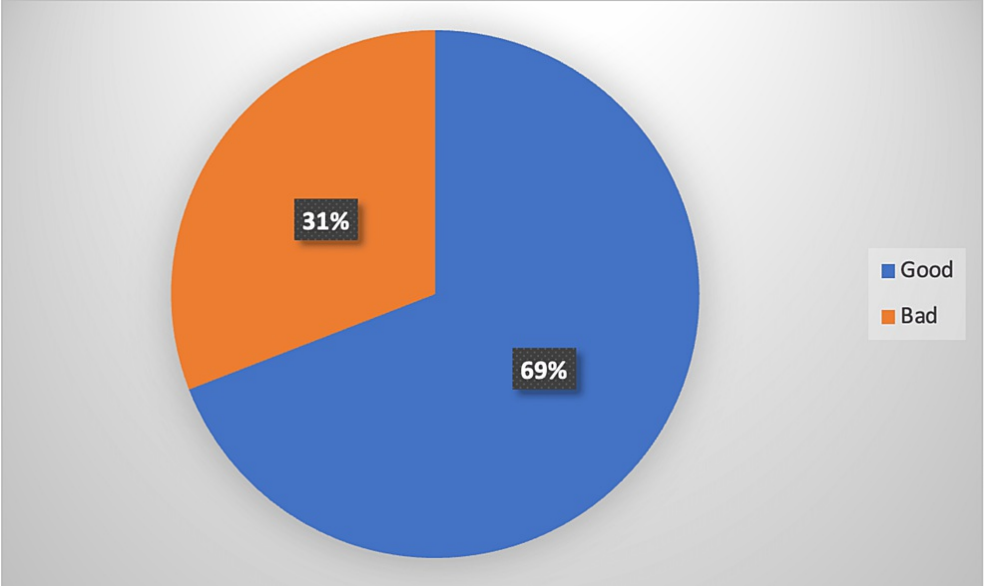
Pie chart depicting the average level of awareness of respondents on various knowledge items. The pie chart above shows there was a moderately good level of knowledge among the respondents (69%).

The pie chart below (Figure 1) shows there was a good level of knowledge among the respondents (69%).

Table 6 below depicts the relationship between social demographic variables and awareness. There was a statistically significant relationship between age and knowledge (p-value = 0.012, p<0.05). There was also a statistical relationship between the level of education and knowledge (p = 0.001). Respondents who had completed at least secondary education had better knowledge compared to others. The other demographic variables did not show any statistically significant differences.

**Table 6:** The association between social demographic variables and levels of awareness.

		Knowledge				
Variables	Category	Good	%	Bad	%	P-value
Sex	Male	80	70.18%	34	29.82%	0.551
	Female	134	67.00%	66	33.00%	
Age	18-24 Years	99	73.88%	35	26.12%	0.012
	25-34 Years	21	65.63%	11	34.38%	
	35-49 Years	45	68.18%	21	31.82%	
	50-65 Years	61	74.39%	21	25.61%	
Marital status	Single/widowed/divorced	95	64.63%	52	35.37%	0.118
	Married	119	71.26%	48	28.74%	
Educational level	Primary	19	63.33%	11	36.67%	0.001
	Middle	17	60.71%	11	39.29%	
	Secondary	55	70.51%	23	29.49%	
	College	137	76.97%	41	23.03%	
The area of residence	Abha	96	66.21%	49	33.79%	0.321
	Khamis Mushait	86	69.35%	38	30.65%	
	Other	30	66.67%	15	33.33%	
Occupation	Medical field	17	89.47%	2	10.53%	0.076
	Non-medical field	61	63.54%	35	36.46%	
	Unemployed	111	55.78%	88	44.22%	
Income	Less than 3000	101	67.79%	48	32.21%	0.111
	3000 to 5000	24	72.73%	9	27.27%	
	5001 to 10000	33	70.21%	14	29.79%	
	More than 10000	59	69.41%	26	30.59%	

## Discussion

This study’s main objective was to determine the awareness of stroke risk factors, warning signs, and preventive behavior among diabetic patients in the Aseer region, of Saudi Arabia. The results of this study indicated that 69.11% of the respondents had good knowledge of stroke, its risk factors, warning signs, and prevention. A good proportion of the participants were aware that the disorder affects the brain (73.89%). This finding is consistent with the study conducted in Al-Ahsa by Elshebiny et al., which found that 61.9% of the respondents knew that stroke affects the brain [9]. Despite the fact that most respondents agreed that stroke is likely to affect the elderly, a large proportion (64.97%) were aware that young people were also at risk.

There was a statistically significant relationship between age and knowledge of stroke (p-value = 0.012, p<0.05). There was also a statistical relationship between the level of education and knowledge (p = 0.001). Respondents who had achieved a college level of education were more knowledgeable. A study by Getu and colleagues conducted in Addis Ababa indicated that a high level of education was a predictor of knowledge of stroke [10]. The respondents demonstrated they had a good level of knowledge of the common risk factors. Most of the respondents asserted that hypertension (74.52%) was the main risk factor for stroke. The respondents also demonstrated an understanding of other factors like smoking, diabetes, and high blood cholesterol. A similar cross-sectional study conducted in Saudi Arabia identified hypertension (81.7%) and family history (74.1%) as the main risk factors [11].

Further, this study revealed that the respondents were aware of the common warning signs of stroke. Most of the respondents identified difficulty speaking (80.57%) and the weakness or inability to move one-half of the body (85.35%) as the dominant warning signs. Hickey et al. found that less than half of the respondents were aware of the warning signs of stroke, especially among the respondents with lower education in Northern Ireland [12]. The findings from Hickey et al. are not consistent with our findings because most of the respondents involved in this study had attained a college level of education.

The high level of awareness of the risk factors, warning signs, and preventive measures does not translate to a reduced incidence of stroke. Sometimes variables like family history can increase susceptibility to stroke. In this study, about 27.71% of the respondents had a family history of stroke. Heredity factors, when combined with unhealthy lifestyles, such as smoking and being obese significantly increase the risk [13].

There is only a handful of prior research on the awareness of stroke risk factors and warning signs among diabetic patients; hence, the literature is limited. A lack of cooperation was observed with some respondents, which made the data collection exercise time-consuming and costly.

## Conclusions

The findings in this study indicated an abstemiously good level of awareness of stroke, its risk factors and warning signs (69.11%). The older respondents and people with a higher level of education demonstrated more knowledge. The two variables, age and education, had a statistically significant relationship with the knowledge of stroke. The common risk factors associated with stroke were identified as hypertension, smoking, diabetes, and high blood cholesterol. The respondents demonstrated good knowledge of the warning signs, with the vast majority asserting that difficulty in speaking, decreased sensation, and weakness or inability to move one-half of the body are common warning signs. Despite respondents showing good knowledge of stroke and its risk factors and warning signs, more awareness is required especially among the elderly group which is at more risk.
